# The M/GP_5_ Glycoprotein Complex of Porcine Reproductive and Respiratory Syndrome Virus Binds the Sialoadhesin Receptor in a Sialic Acid-Dependent Manner

**DOI:** 10.1371/journal.ppat.1000730

**Published:** 2010-01-15

**Authors:** Wander Van Breedam, Hanne Van Gorp, Jiquan Q. Zhang, Paul R. Crocker, Peter L. Delputte, Hans J. Nauwynck

**Affiliations:** 1 Laboratory of Virology, Department of Virology, Parasitology and Immunology, Faculty of Veterinary Medicine, Ghent University, Merelbeke, Belgium; 2 Division of Cell Biology and Immunology, Wellcome Trust Biocentre, University of Dundee, Dundee, United Kingdom; University of Washington, United States of America

## Abstract

The porcine reproductive and respiratory syndrome virus (PRRSV) is a major threat to swine health worldwide and is considered the most significant viral disease in the swine industry today. In past years, studies on the entry of the virus into its host cell have led to the identification of a number of essential virus receptors and entry mediators. However, viral counterparts for these molecules have remained elusive and this has made rational development of new generation vaccines impossible. The main objective of this study was to identify the viral counterparts for sialoadhesin, a crucial PRRSV receptor on macrophages. For this purpose, a soluble form of sialoadhesin was constructed and validated. The soluble sialoadhesin could bind PRRSV in a sialic acid-dependent manner and could neutralize PRRSV infection of macrophages, thereby confirming the role of sialoadhesin as an essential PRRSV receptor on macrophages. Although sialic acids are present on the GP_3_, GP_4_ and GP_5_ envelope glycoproteins, only the M/GP_5_ glycoprotein complex of PRRSV was identified as a ligand for sialoadhesin. The interaction was found to be dependent on the sialic acid binding capacity of sialoadhesin and on the presence of sialic acids on GP_5_. These findings not only contribute to a better understanding of PRRSV biology, but the knowledge and tools generated in this study also hold the key to the development of a new generation of PRRSV vaccines.

## Author Summary

The porcine reproductive and respiratory syndrome virus (PRRSV) is a major threat to swine health worldwide. The virus specifically targets subpopulations of macrophages, central players in the immune system, and can persist in animals for extended periods of time due to a hampered immunity. At present, no vaccines are available that are both safe and effective and it is clear that a more rational vaccine design is needed to solve this problem. Therefore, advancing our fundamental understanding of PRRSV biology is crucial. The macrophage-specific lectin sialoadhesin is a crucial viral receptor on macrophages and although its role in PRRSV infection is well documented, its viral counterparts have remained unknown. Using a soluble form of sialoadhesin, we identified the M/GP_5_ glycoprotein complex of PRRSV as the ligand for sialoadhesin and found this ligand-receptor interaction to be critically dependent on the lectin activity of sialoadhesin and on sialic acids on the GP_5_ glycoprotein. These data represent a major breakthrough in the understanding of the role of PRRSV proteins in viral entry and pave the way for the development of a new generation of PRRSV vaccines capable of inducing an immunity that specifically blocks the interaction between viral M/GP_5_ and sialoadhesin.

## Introduction

At the end of the 1980s, a new syndrome was described affecting pig herds in North America and Canada [1],[2]. This ‘mystery swine disease’ manifested itself in respiratory problems and reproductive disorders and was eventually designated Porcine Reproductive and Respiratory Syndrome (PRRS), reflecting the associated disease symptoms. The causative agent is a positive sense RNA virus that groups within the order *Nidovirales*, family *Arteriviridae*, and is referred to as the PRRS virus (PRRSV) [3]. At present, the disease is endemic in swine-producing countries worldwide, causing enormous production losses in the swine industry. A study by Neumann *et al.* assessing the economic impact of PRRS on swine production in the US reported an annual loss of approximately 560.32 million US dollars due to this syndrome [4]. Also, recent studies report on the emergence of highly pathogenic variants of the virus in Asia causing atypical PRRS or ‘High Fever’ disease [5]–[7]. Consequently, PRRS is considered to be the most significant viral disease problem in the swine industry today.

Availability of safe and effective vaccines is essential for PRRSV control. Currently, there are two types of PRRSV vaccines on the market: attenuated and inactivated vaccines. However, these have specific drawbacks concerning safety [8]–[10] and efficacy [11]–[15] and there is a strong demand for a new generation of vaccines. Up until now, PRRSV vaccine development often has followed the trial and error approach. As there was a clear gap in the knowledge of PRRSV ligands that bind to specific receptors, it was difficult to aim for specific blocking of crucial steps in PRRSV infection of the porcine macrophage. A fundamental understanding of how PRRSV enters its host cell is crucial to reverse the tide.

The PRRSV virion consists of a nucleocapsid that is surrounded by a lipid envelope. The capsid structure consists of nucleocapsid proteins (N) and contains the viral genome: a single, positive RNA strand of approximately 15 kb [16]–[21]. In the viral envelope, six structural proteins are embedded: the small envelope protein E, the membrane protein M and the glycoproteins GP_2_, GP_3_, GP_4_ and GP_5_ [21]. However, some North American PRRSV isolates do not seem to incorporate GP_3_ as a structural membrane protein, in contrast to European and other North American isolates [21]–[24]. The major structural proteins M and GP_5_ have been shown to form disulfide-linked heterodimers [21],[25],[26]. The minor structural proteins GP_2_, GP_3_ and GP_4_ form non-covalent heterotrimers in the virion and there are indications that also the E protein may be associated with the minor glycoprotein trimer [21], [23], [27]–[29].

As has been shown for other arteriviruses, PRRSV shows a marked *in vivo* tropism for cells of the monocyte/macrophage lineage: the virus infects specific subsets of porcine macrophages [30],[31]. Primary cultures of alveolar macrophages (PAM) are the only cells that allow efficient *ex vivo* virus propagation. In addition, a limited number of cell lines support *in vitro* virus replication upon adaptation of the virus. One such cell line, the African green monkey kidney cell line MARC-145 [32], has become the most widely used cell type for PRRS virus production.

Over the past years, various studies have focussed on the entry of PRRSV into its host cell. These efforts have resulted in the identification of a number of macrophage molecules involved in PRRSV entry. As for many other viruses, initial binding of the virus to its host cell occurs via interactions with heparan sulphate glycosaminoglycans present on the cell surface [33]–[35]. The virus receptor that determines subsequent virus entry and that likely accounts for the macrophage tropism of PRRSV has been identified as porcine sialoadhesin (pSn). This macrophage-specific molecule is a sialic acid-binding immunoglobulin-like lectin (siglec) that mediates virus attachment and subsequent internalization via clathrin-mediated endocytosis [36],[37]. Virus attachment to this receptor is dependent on the sialic acid-binding activity of the N-terminal immunoglobulin-like domain of pSn [38] and on the presence of sialic acids on the virion surface [39]. A study by Delputte *et al.* pointed out that α2-3 linked sialic acids and to a lesser extent α2-6 linked sialic acids, most likely present on complex N-linked glycans attached to viral envelope glycoproteins, are involved in this interaction [39]. Clearly, a glycosylated PRRSV protein is responsible for PRRSV binding to pSn, but the exact viral ligand has not yet been identified. After pSn-dependent internalization into the endosomal compartment of the macrophage, the viral genome is released into the cytoplasm, thereby initiating the transcriptional and translational events necessary for the production of new virions. The scavenger receptor CD163 has been shown to be essential for virus uncoating [40],[41]. A pH drop within the endosome is required [37],[42] and also the aspartic protease cathepsin E and a yet unidentified trypsin-like serine protease [43] have been implicated in this process.

Although pSn has been shown to be a critical entry receptor for PRRSV on macrophages, viral envelope glycoproteins that act as ligands for this receptor have remained elusive. In the light of vaccine development, knowledge on this is particularly interesting, since it allows targeting of the immune response to a specific, critical step in virus infection. Vaccination with a functional viral pSn-binding epitope can elicit a protective immune response that specifically blocks the crucial, pSn-dependent internalization of the virus into its host cell. By construction and use of soluble recombinant porcine sialoadhesins, we identified the viral M/GP_5_ glycoprotein complex as a ligand for pSn and showed the sialic acid-dependency of the pSn-M/GP_5_ interaction.

## Materials and Methods

### Ethics statement

The experimental procedure for the collection of porcine alveolar macrophages was authorized and supervised by the Ethical and Animal Welfare Committee of the Faculty of Veterinary Medicine of Ghent University. The use of human red blood cells was approved by the Medical Ethical Committee of the Ghent University Hospital and informed written consent was obtained from the donors of red blood cells.

### Monoclonal antibodies

Detection of pSn was performed using the mouse monoclonal antibody (mAb) 41D3 [36],[44] that recognizes a conformational epitope within the N-terminal sialic acid-binding domain of pSn. Detection of structural PRRSV proteins was performed using the following mAbs: mAb VII2D/5-1D (IgG1), mAb XVI11C/5-10F (IgG2a) and mAb VII2H/2-4D (IgG1) [45] were used for the detection of GP_3_, GP_4_ and GP_5_, respectively. Detection of the M protein was performed using mAb 126.3 (IgG2a) [28] and mAb P3/27 (IgG1) [46] was used for detection of the N protein. MAb 13D12 [47] and mAb 16G12 [48] were used as isotype-matched, irrelevant control mAbs for the mAbs with IgG1 and IgG2a isotype, respectively.

### Cells and viruses

Porcine alveolar macrophages (PAM) were obtained from 4- to 6-week-old conventional Belgian Landrace pigs from a PRRSV-negative herd as described by Wensvoort *et al.* [2] and cultivated as described by Van Gorp *et al.* [40]. MARC-145 cells were cultivated as described before [40]. HEK-293T cells were grown in DMEM (Gibco) containing 10% heat-inactivated FBS (Gibco), 2 mM L-glutamine, 1 mM sodium pyruvate and a mixture of antibiotics. Cell cultures were kept in a humidified 5% CO_2_ atmosphere at 37°C.

Human red blood cells (RBCs) were obtained from healthy donors and stored at 4°C in Alsever's solution for up to 7 days.

The European prototype PRRSV strain Lelystad Virus [2] (LV; kindly provided by G. Wensvoort) was passaged 14 times on macrophages (macrophage-grown LV stock) or 13 times on macrophages and subsequently 5 times on MARC-145 cells (MARC-145-grown LV stock). Virus was semipurified from the supernatants via ultracentrifugation as described before [39]. Virus titrations on MARC-145 cells and macrophages and calculation of the virus titers were performed as described by Van Gorp *et al.* [40].

### Construction, production and purification of pSn-Fc fusion proteins

The pSn cDNA had been cloned previously into the pcDNA3.1/D vector (Invitrogen) [36]. The soluble Fc-tagged pSn, pSn4D-Fc, was generated by polymerase chain reaction (PCR) amplification as done before for human sialoadhesin [49] and cloning into a modified version of the pEE14 vector, designated pEE14-3C-Fc [50]. The cDNA fragment corresponding to the first 4 N-terminal immunoglobulin-like domains of pSn was amplified using forward primer 5′-CCTTCACCATGGACTTCCTG-3′ and reverse primer 5′-ACTAGATCTACTTACCTGTGCTGACCACCACGCTGACAG-3′. The pEE14-3C-Fc vector was cut with HindIII, treated with Klenow DNA polymerase to obtain blunt ends and subsequently cut with BamHI. The PCR product was digested with BglII and cloned into the cut pEE14-3C-Fc vector, yielding pEE14-pSn4D-3C-Fc. To obtain a non-sialic acid-binding pSn4D-Fc protein, a point-mutation (R^116^E) was introduced in the sialic acid-binding domain in pEE14-pSn4D-3C-Fc using the Quickchange site directed mutagenesis kit (Stratagene) with forward primer 5′-TCGGGCTCCTATAACTTCgaaTTTGAGATCAGCGAGGGC-3′ and reverse primer 5′-GCCCTCGCTGATCTCAAAttcGAAGTTATAGGAGCCCGA-3′, resulting in pEE14-pSn4D_RE_-3C-Fc.

For production of the pSn-Fc chimeras, HEK-293T cells were transiently transfected using calcium phosphate. Transfected cells were cultured for 3 days in DMEM supplemented with 3% IgG-depleted low IgG FBS (Gibco), 2 mM L-glutamine, 1 mM sodium pyruvate, 1% nonessential amino acids (100× stock; Gibco) and a mixture of antibiotics in a humidified 5% CO_2_ atmosphere at 37°C, after which the culture supernatant was collected. The pSn-Fc fusion proteins were purified from the supernatant using standard protein A sepharose chromatography following the manufacturer's instructions (GE Healthcare). Fractions of the eluate containing the purified protein were pooled and the buffer was exchanged to phosphate-buffered saline (PBS) by dialysis. Purified protein was stored at −70°C until use.

### Solid phase red blood cell binding assay

Each well of an Immulon 4HBX 96-well flat-bottomed microtiter plate (Dynax Technologies Inc.) was coated with 50 µl of 8 µg/ml goat anti-human IgG (Fc- specific; Sigma-Aldrich Corp.) in 0.05 M carbonate-bicarbonate buffer (pH 9.6; Sigma-Aldrich Corp.). Plates were washed with PBS containing 0.25% bovine serum albumin (PBA), after which 2-fold dilution series of the purified siglec-Fc chimera were added to the wells and incubated for 2 h at ambient temperature. Unbound siglec-Fc protein was removed by washing with PBA. RBCs were washed twice in PBA and resuspended in PBA at 0.25% (v/v) immediately before use. 100 µl of the RBC suspension was added to each well and the plate was incubated for 30 min at 37°C. The wells were gently washed with PBA to remove unbound RBCs and RBC binding was evaluated using an Olympus CK40-F200 inverted light microscope (Opelco). To quantify RBC binding, the plate was dried, fixed with methanol and dried again, after which a peroxidase substrate assay was performed (Substrate Reagent Pack; R&D Systems; processed according to manufacturer's instructions). The result was evaluated measuring the optical density at 450 nm (OD450) using a Multiskan RC (Thermo Labsystems). As a positive control, an identical assay was performed in parallel for mSiglecE-Fc [51], a protein with established sialic acid-binding activity. As a control for sialic acid-dependent binding, sialidase-treated RBCs were used. To remove sialic acids from the cell surface, RBCs were diluted (0.25% v/v) in RPMI-1640 containing 50 mU/ml *Vibrio cholerae* sialidase (Roche Applied Science; specific for α2-3, 6, 8 linked sialic acids) and incubated for 1 h at 37°C. The RBCs were subsequently washed with PBA to remove the enzyme, resuspended in PBA at 0.25% (v/v) and used in the solid phase binding assay as described.

### SDS-PAGE, Coomassie Blue staining and Western Blot analysis of pSn-Fc proteins

Samples of purified pSn-Fc protein were mixed with (non-) reducing Laemmli buffer, boiled for 5 min and subjected to SDS-PAGE (8% gel) using a BioRad Mini Protean 3 system. For Coomassie Blue staining, the SDS-PAGE gel was incubated successively in Coomassie Blue staining solution (0.125% Coomassie Blue, 50% methanol, 10% acetic acid), destaining solution I (50% methanol, 10% acetic acid) and destaining solution II (5% methanol, 7% acetic acid). Alternatively, for Western Blot analysis, proteins were transferred from the SDS-PAGE gel to a PVDF membrane (Membrane Hybond-P, GE Healthcare) via Western Blotting (BioRad Mini Trans Blot). The membrane was blocked overnight in PBS +0.1% Tween 20+5% skimmed milk. Detection of pSn-Fc protein was performed by subsequent incubation of the blot with the pSn-specific mAb 41D3 and peroxidase-labeled polyclonal goat anti-mouse antibodies (Dako), followed by visualization using enhanced chemiluminiscence (ECL; GE Healthcare). Alternatively, pSn-Fc protein was detected using peroxidase-labeled goat anti-human IgG antibodies (Fc-specific; Sigma-Aldrich Corp.) and subsequent ECL.

### Analysis of intracellular processing of pSn-Fc proteins

Samples of purified pSn4D-Fc and pSn4D_RE_-Fc protein were either left untreated or were treated with endoglycosidase H or PNGase F (New England Biolabs Inc.; used according to manufacturer's instructions). Also, samples of both proteins were incubated with 100 mU/ml *Vibrio cholerae* sialidase (Roche Applied Science; used according to manufacturer's instructions) for 3 h at 37°C. After addition of reducing Laemmli buffer, samples were boiled for 5 min and analyzed via SDS-PAGE and Western Blot using peroxidase-labeled goat anti-human IgG antibodies as described above.

### Evaluation of the PRRSV-binding capacity of pSn4D-Fc via precipitation experiments

100 µl of Dynabeads protein A (Invitrogen) were coated with 25 µg of pSn4D-Fc protein and incubated with semipurified, MARC-145-grown PRRSV LV at 37°C. After 90 min of incubation, the unbound virus fraction was collected, beads were washed 4 times with PBS and bound material was eluted with 0.1 M citrate buffer (pH 3.1). Samples of the semipurified virus, the bound and the unbound fraction were mixed with non-reducing Laemmli buffer, boiled for 5 min and resolved on a 12% SDS-PAGE gel. Subsequently, resolved proteins were transferred to a PVDF membrane via Western Blotting. The membrane was blocked overnight in PBS +0.1% Tween 20+5% skimmed milk and probed with a nucleocapsid-specific, a GP_5_-specific or an isotype-matched irrelevant control mAb in combination with peroxidase-labeled polyclonal goat anti-mouse antibodies. The detected protein bands were visualized using ECL. To confirm that the beads were efficiently coated with pSn-Fc protein, a sample of the bound fraction was subjected to SDS-PAGE and Western Blot analysis using the pSn-specific mAb 41D3. As a control, identical experiments were performed using uncoated beads and beads coated with the non-sialic acid-binding mutant pSn4D_RE_-Fc.

### Evaluation of the PRRSV-binding capacity of pSn4D-Fc via infection-inhibition experiments

PAM were seeded at a concentration of 10^5^ cells/well in 96-well plates and kept in culture for 48 h. 50 µl of a 3-fold dilution series of pSn4D-Fc or pSn4D_RE_-Fc (starting concentration 50 µg/ml) was mixed with a constant amount of virus (5 µl of a 2×10^5^ TCID_50_/ml virus suspension) and incubated for 1 h at 37°C to allow binding. The mixtures were transferred to the PAM and cells were incubated for 1 h at 37°C. Soluble receptor-virus mixtures were then removed, fresh medium was added and cells were further incubated for 9 h at 37°C, after which they were fixed. Infected cells were then visualized via a nucleocapsid-specific immunoperoxidase staining [2] and counted.

### Identification of pSn-binding PRRSV proteins and characterization of pSn-ligand interaction using pSn4D-Fc

Viral proteins were solubilized from semipurified MARC-145-grown PRRSV LV by a 1 h incubation in TNE buffer (50 mM Tris-HCl (pH 7.4), 200 mM NaCl, 1 mM EDTA) containing 1% NP-40 (Roche Applied Science) and insoluble material was pelleted by centrifugation at 10,000×g for 45 min. Subsequently, the virus lysate was incubated with pSn4D-Fc-coated beads. The precipitation experiment, sample preparation and subsequent SDS-PAGE and Western Blotting were performed as described above. Membranes were blocked overnight in PBS +0.1% Tween 20+5% skimmed milk and probed with mAbs directed against the structural proteins of PRRSV LV and isotype-matched irrelevant control mAbs in combination with peroxidase-labeled polyclonal goat anti-mouse antibodies. Detected protein bands were visualized using ECL. Coating of the beads with pSn-Fc protein was checked as described above. To assess the sialic acid-dependency of the interaction, an identical experiment was performed using the non-sialic acid-binding mutant pSn4D_RE_-Fc. Alternatively, sialidase-treated virus was used in the precipitations. Semi-purified virus was incubated with 100 mU/ml sialidase from *Vibrio cholerae* in RPMI-1640 for 3 h at 37°C. In parallel, virus was incubated with the buffer in which the sialidase was supplied. Titration on alveolar macrophages revealed a 90% decrease in infectivity of sialidase-treated virus when compared to control-treated virus, indicating that sialidase treatment removed sialic acids implicated in viral entry. To evaluate the effect of the treatment on different envelope glycoproteins, samples of sialidase- and control-treated virus were subjected to SDS-PAGE and Western Blot analysis using specific mAbs. The remaining virus was lysed and subjected to immunoprecipitation using pSn4D-Fc as described above.

Similar precipitation reactions were performed using macrophage-grown virus. Lysates of semipurified macrophage-grown virus were applied to pSn4D-Fc- and pSn4D_RE_-Fc-coated beads. The bound and unbound fractions as well as the original virus lysates were analyzed via SDS-PAGE and Western Blotting as described above.

### Accession numbers

GenBank accession numbers (http://www.ncbi.nlm.nih.gov/Genbank):

- PRRSV Lelystad virus, complete genome (coding sequence): M96262
- PRRSV Lelystad virus, individual structural proteins (protein): AAA46275 (GP2); AAA46276 (GP3); AAA46277 (GP4); AAA46278 (GP5); AAA46279 (M); AAA46280 (N); P0C6Y6 (E)
- Porcine sialoadhesin: AF509585 (mRNA, complete cds), AAP47136 (protein)
- Porcine CD163: EU016226 (mRNA, complete cds), ABV80230 (protein)
- Murine siglec E: AY371487 (mRNA, complete cds), AAQ72479 (protein)

## Results

### Construction and structural characterization of pSn-Fc chimeras

To identify the PRRSV envelope glycoproteins interacting with pSn, we constructed a soluble form of the pSn receptor (pSn4D-Fc). This recombinant protein consists of the 4 N-terminal domains of pSn, coupled with the Fc- and hinge region of human IgG1 and allows study of PRRSV-pSn interactions in a cell-free context. The sialic acid-binding activity of pSn has been shown before to be essential for PRRSV binding [38]. Therefore, a non-sialic acid-binding mutant of the soluble pSn (pSn4D_RE_-Fc) was generated as a control. This protein was obtained by changing the amino acid R^116^, which is essential for sialic acid binding, to an E residue. PSn- and Fc-specific immunofluorescence cell stainings (Protocol S1) and ELISAs of the cell supernatants (Protocol S2) revealed that the pSn-Fc chimeras were expressed in transfected HEK-293T cells and that they were secreted into the culture medium (data not shown).

**Figure 1:**
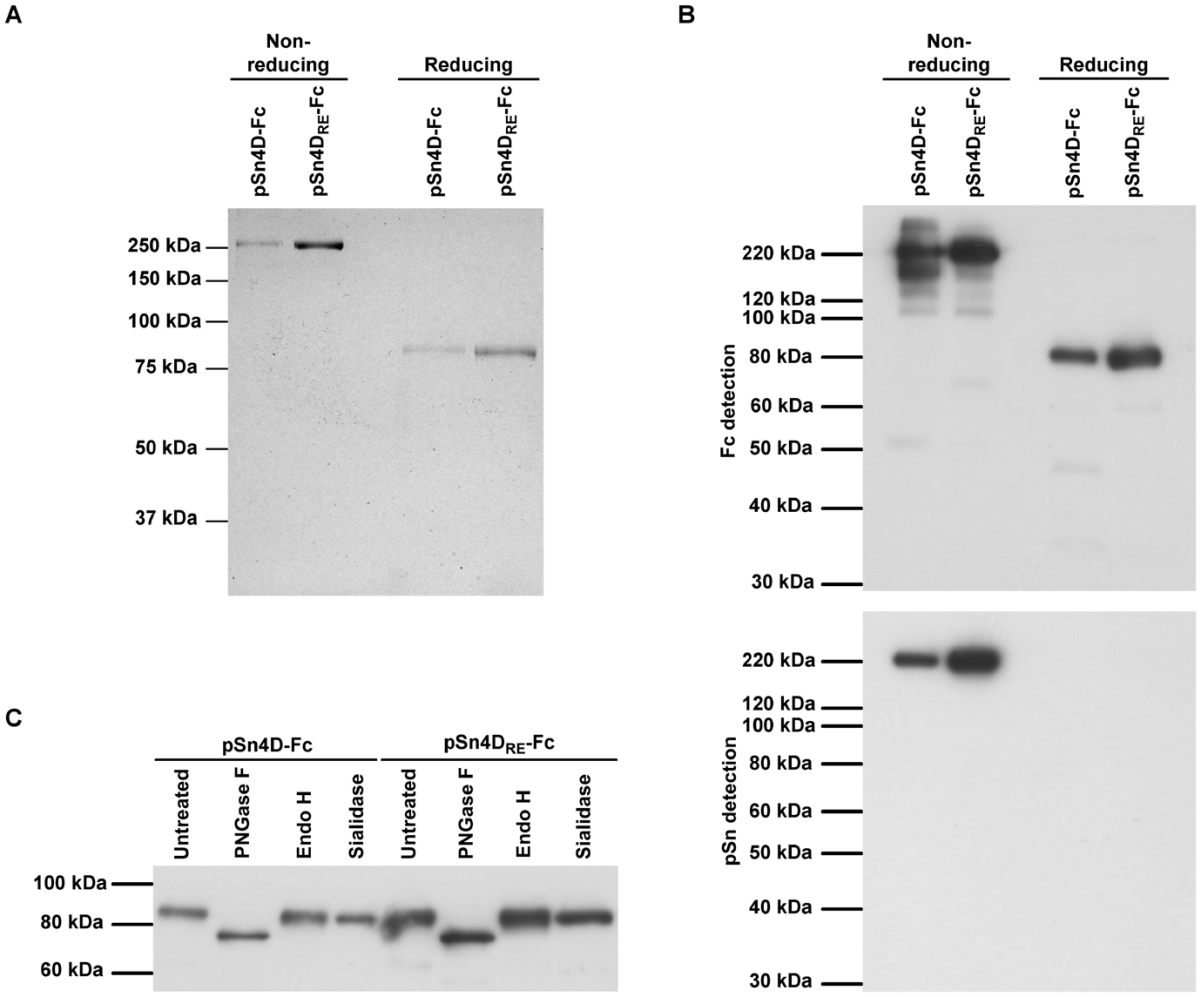
Structural characterization of pSn-Fc proteins. (**A**) Samples of purified pSn4D-Fc and pSn4D_RE_-Fc proteins were subjected to non-reducing and reducing SDS-PAGE, after which Coomassie Blue staining was performed. (**B**) (Non-)reducing SDS-PAGE and Western Blot analysis of purified pSn4D-Fc and pSn4D_RE_-Fc proteins using Fc-specific antibodies and pSn-specific mAb 41D3. (**C**) pSn4D-Fc and pSn4D_RE_-Fc proteins were treated with specific glycosidases or control treated and subjected to reducing SDS-PAGE and Western Blot analysis using Fc-specific antibodies.

After optimization of production and purification, all further tests and experiments were performed using purified pSn-Fc proteins. To check the purity of the proteins, samples were resolved on SDS-PAGE under reducing and non-reducing conditions and Coomassie Blue staining was performed (Fig. 1A). Under non-reducing conditions, a single band of more than 250 kDa was seen for both the pSn4D-Fc and pSn4D_RE_-Fc protein. Presence of a reducing agent resulted in single bands at about 80 kDa. These results indicate that the purified pSn-Fc proteins are present as disulfide-linked dimers under non-reducing conditions. The fact that single bands were obtained under each condition shows that the bulk of the protein in the purified fractions was pSn-Fc protein.

Purified proteins were also subjected to SDS-PAGE and Western Blot analysis (Fig. 1B). Under non-reducing conditions, both pSn4D-Fc and pSn4D_RE_-Fc were recognized by pSn-specific mAb 41D3. When SDS-PAGE was performed in the presence of a reducing agent, binding of mAb 41D3 to the blotted proteins was lost, which is in line with earlier observations for wild type pSn. Both the pSn4D-Fc and the pSn4D_RE_-Fc proteins could be detected on the blot membrane using polyclonal antibodies specific for the Fc-part of human IgG. Using the Fc-specific polyclonal antibodies for detection, additional bands could be observed with varying intensity in between batches. These bands most likely represent pSn-Fc protein that is partially degraded. Nevertheless, Coomassie Blue analysis of the same samples clearly indicated that the bulk of the protein was present in one specific band, which correlates with conformationally correct pSn-Fc protein.

Wild type pSn undergoes processing in the endoplasmic reticulum (ER) and Golgi before being displayed at the cell membrane. During its passage through these compartments, the protein does not only obtain intramolecular disulfide bridges, but also N-linked glycans, which are often essential for proper protein folding. To check intracellular processing of the recombinant proteins, purified pSn4D-Fc and pSn4D_RE_-Fc proteins were treated with different glycosidases and analyzed via reducing SDS-PAGE and Western Blotting (Fig. 1C). Treatment of the proteins with N-glycosidase F, which removes all types of N-linked glycans, resulted in a clear shift in the protein size. Treatment with endoglycosidase H, which removes high mannose and some hybrid types of N-linked carbohydrates from glycoproteins, or *Vibrio cholerae* sialidase, removing sialic acids in a α2-3, α2-6 or α2-8 configuration, did not increase the electrophoretic mobility of the proteins. These results indicate that the soluble pSn proteins carry mainly complex type N-glycans capped with little or no sialic acids. The presence of these sugar moieties shows that the soluble sialoadhesins pass through the ER and Golgi for processing, as has been shown for wild type pSn [36].

### Functional characterization of pSn-Fc chimeras

**Figure 2:**
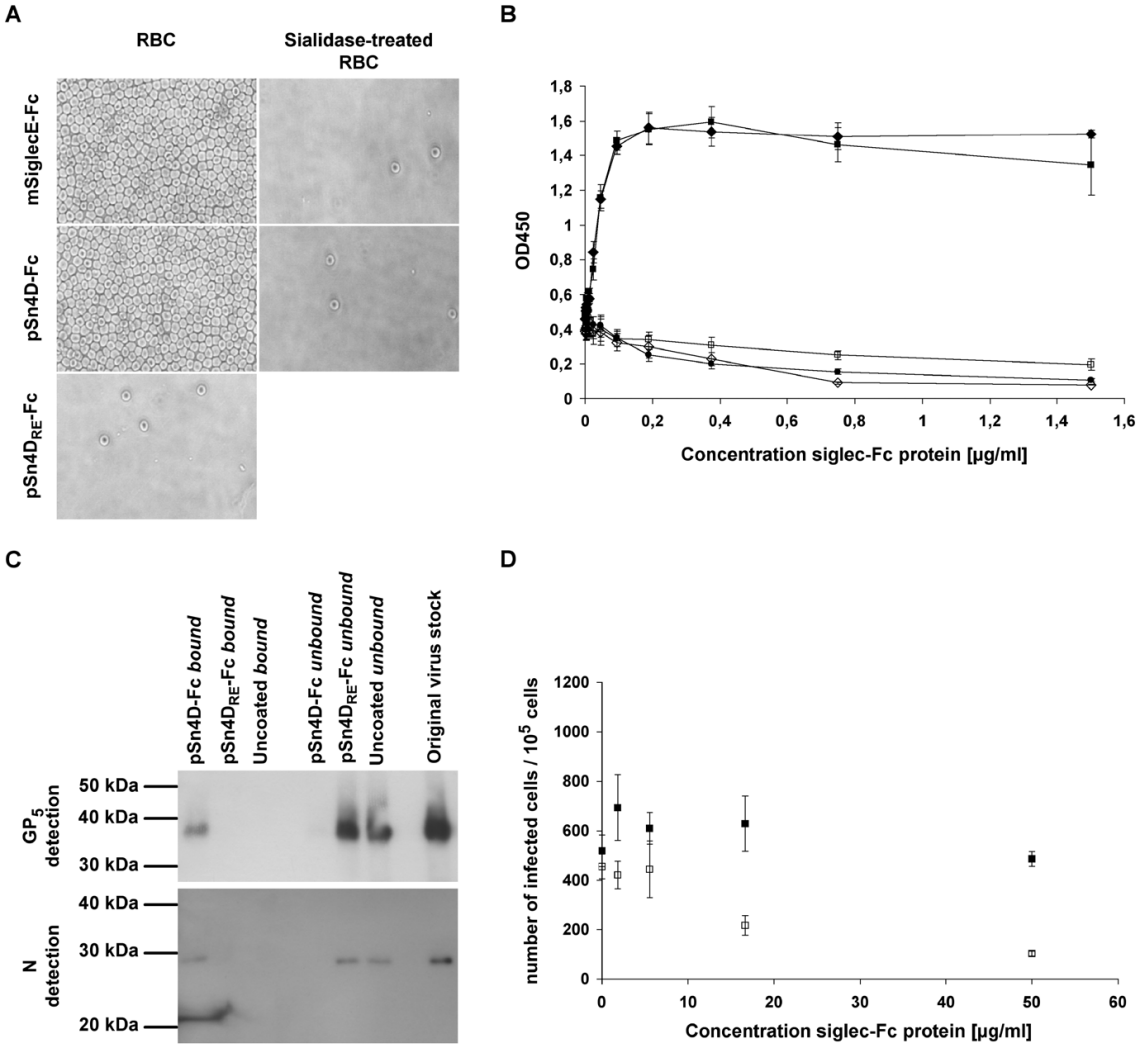
Evaluation of the functionality of pSn4D-Fc as a sialic acid-binding (A & B) and PRRSV-binding (C & D) protein. Qualitative (**A**) and quantitative (**B**) analysis of sialic acid-binding activity using a solid phase red blood cell binding assay. ELISA plates were coated with dilution series of pSn4D-Fc, pSn4D_RE_-Fc or mSiglecE-Fc protein, after which human RBCs were added and RBC binding was evaluated. pSn4D-Fc + RBCs (black square); pSn4D-Fc + sialidase-treated RBCs (white square); mSiglecE-Fc + RBCs (black diamond); mSiglecE-Fc + sialidase-treated RBCs (white diamond); pSn4D_RE_-Fc + RBCs (black circle). Values represent means±SEM of 3 experiments. (**C**) Evaluation of PRRSV-binding activity via immunoprecipitation experiments. Protein A beads were either coated with the pSn4D-Fc or pSn4D_RE_-Fc protein or not coated and incubated with MARC-145-grown PRRSV at 37°C to allow binding. The bound and unbound fractions were collected and subjected to non-reducing SDS-PAGE and Western Blot analysis using PRRSV N- and GP_5_-specific mAbs. (**D**) Evaluation of PRRSV-binding activity via infection-inhibition experiments. A 3-fold dilution series of pSn-Fc protein was mixed with a constant amount of MARC-145-grown PRRSV, incubated for 1 h at 37°C to allow binding and transferred to 10^5^ alveolar macrophages. After 1 h of incubation at 37°C, pSn-Fc - virus mixtures were removed, fresh medium was added to the cells and cells were incubated for 9 h at 37°C, after which they were fixed. Infected cells were then visualized via nucleocapsid-specific immunoperoxidase staining and counted. pSn4D-Fc (white square); pSn4D_RE_-Fc (black square). Values represent means±SEM of 3 experiments.

Since the sialic acid-binding activity of pSn is essential for PRRSV binding, we first evaluated the sialic acid-binding capacity of the purified pSn4D-Fc protein. This was done via a solid phase red blood cell binding assay, a test routinely used to analyze the sialic acid-binding capacity of sialoadhesin-Fc and other siglec-Fc chimeras [52] (Fig. 2A & 2B). The pSn4D-Fc protein showed clear, sialic acid-dependent RBC binding, since removal of sialic acids from the RBC surface impeded the interaction. The pSn4D_RE_-Fc protein did not show RBC-binding activity. These results indicate that pSn4D-Fc has the capacity to bind sialic acids. As for full length pSn, the sialic acid-binding activity is critically dependent on the R^116^ residue within the N-terminal domain of pSn.

To assess the PRRSV-binding capacity, pSn4D-Fc was coated on protein A beads and incubated with purified virus at 37°C to allow binding. The unbound fraction was then collected, beads were washed and the bound material was eluted from the beads. Samples of the bound and unbound fraction and samples of purified virus were then subjected to non-reducing SDS-PAGE and Western Blotting and analyzed for the presence of virus using mAbs recognizing the GP_5_ envelope glycoprotein and the nucleocapsid protein N (Fig. 2C). GP_5_ and N could clearly be detected in both the purified virus stock and the bound fraction, while little or no protein was found in the unbound fraction. These results show that pSn4D-Fc is able to efficiently bind the virus. When beads were coated with pSn4D_RE_-Fc or when uncoated beads were used, viral proteins were detected in the unbound fraction but not in the bound fraction. These findings show that the interaction of pSn4D-Fc with PRRSV is similar to the interaction between wild type pSn and the virus. To further substantiate this, we tested the soluble receptors for their infection-inhibition capacity (Fig. 2D). We found that PRRSV infection of alveolar macrophages could be partially blocked by pre-incubating the virus with pSn4D-Fc. This effect was dose-dependent and no such effect was obtained using the pSn4D_RE_-Fc protein, indicating that also here the sialic acid-binding functionality of pSn is crucial for the interaction. These data evidence that the soluble sialoadhesin pSn4D-Fc can compete with the wild type pSn on the macrophage surface for specific ligands present on the PRRSV virion surface and confirm the role of pSn as an important PRRSV receptor on macrophages.

### Identification of pSn-binding PRRSV proteins and characterization of the pSn-ligand interaction

**Figure 3:**
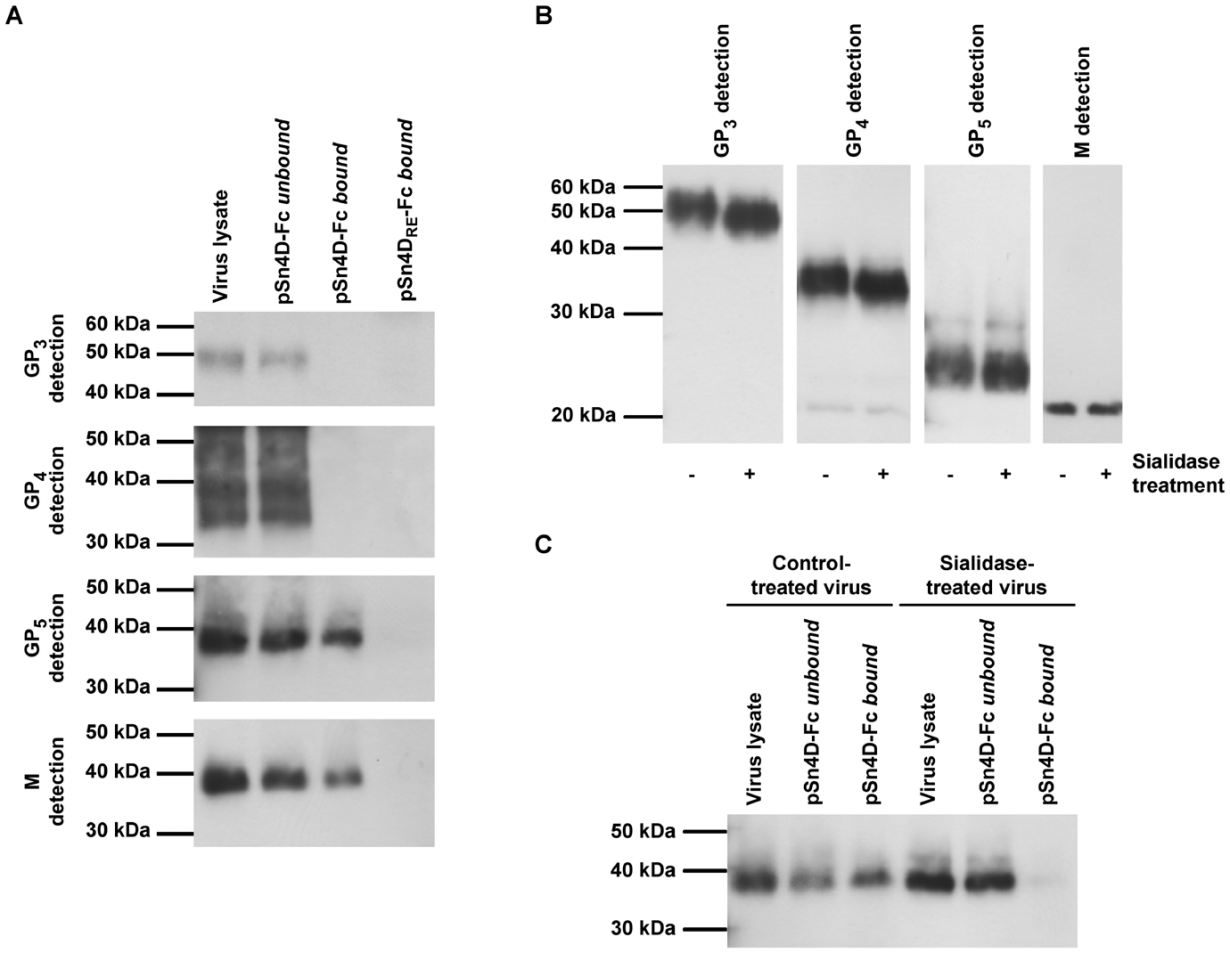
Identification of pSn-binding (glyco)protein(complexe)s of MARC-145-grown PRRSV and characterization of the pSn-ligand interaction. (**A**) Protein A beads were coated with the pSn4D-Fc or pSn4D_RE_-Fc proteins and incubated with a lysate of MARC-145-grown PRRSV at 37°C to allow binding. The bound and unbound fractions were collected and subjected to non-reducing SDS-PAGE and Western Blot analysis using virus-specific mAbs. (**B**) MARC-145-grown PRRSV was either treated with sialidase or control-treated and subjected to reducing SDS-PAGE and Western Blot analysis using virus-specific mAbs. (**C**) MARC-145-grown PRRSV was either treated with sialidase or control-treated, lysed and incubated with pSn4D-Fc-coated protein A beads at 37°C to allow binding. The bound and unbound fractions were collected and subjected to non-reducing SDS-PAGE and Western Blot analysis using a PRRSV GP_5_-specific mAb.

To identify viral ligands for the PRRSV receptor pSn, pSn4D-Fc was used in an immunoprecipitation reaction. PSn4D-Fc was coated on protein A beads and incubated with a lysate of purified virus at 37°C to allow binding. Subsequently, the unbound lysate fraction was collected, beads were washed and the bound material was eluted from the beads. Samples of the bound and unbound fraction and samples of the original virus lysate were subjected to non-reducing SDS-PAGE and Western Blotting and analyzed for the presence of specific viral envelope proteins using protein-specific mAbs (Fig. 3A). GP_3_, GP_4_ and the M/GP_5_ complex were all detected in the original virus lysate and in the unbound fraction. In addition, the M/GP_5_ complex was detected in the bound fraction. GP_3_ and GP_4_ were however not found in the bound fraction. These results indicate that the viral M/GP_5_ complex is able to bind to pSn4D-Fc. Control experiments with pSn4D_RE_-Fc pointed out that this protein does not interact with any of the viral proteins analyzed. This shows that the sialic acid-binding capacity of pSn is essential for M/GP_5_ binding and suggests that sialic acids on GP_5_ are involved in the interaction with pSn. Sialidase treatment of PRRSV, which removes sialic acids from the GP_5_ protein as indicated by an increased electrophoretic mobility of the protein on SDS-PAGE (Fig. 3B), indeed also resulted in loss of M/GP_5_ binding to pSn4D-Fc (Fig. 3C).

**Figure 4:**
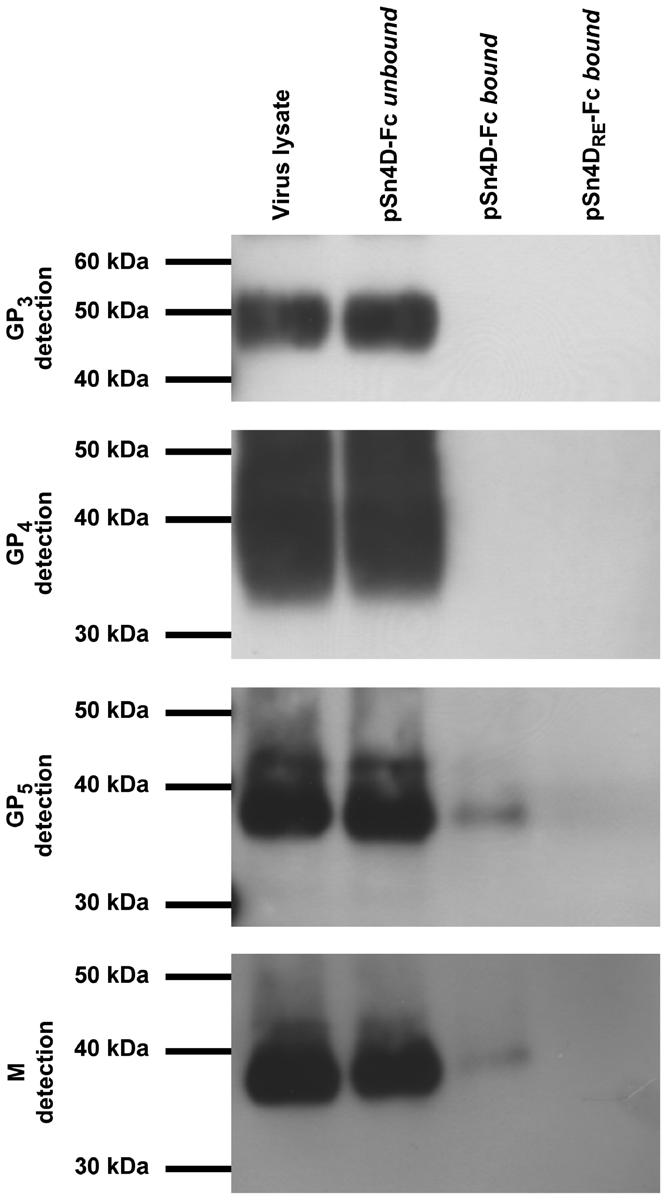
Identification of pSn-binding (glyco)protein(complexe)s of macrophage-grown PRRSV. Protein A beads were coated with the pSn4D-Fc or pSn4D_RE_-Fc protein and incubated with a lysate of macrophage-grown PRRSV at 37°C to allow binding. The bound and unbound fractions were collected and subjected to non-reducing SDS-PAGE and Western Blot analysis using virus-specific mAbs.

The experiments described above clearly showed that sialic acids on the envelope glycoproteins are essential for the pSn-M/GP_5_ interaction. However, as the glycosylation machinery of MARC-145 cells and porcine macrophages differs, their glycome and the glycan array present on virus grown in these cells may differ substantially. As all experiments were performed with MARC-145-grown virus, immunoprecipitations were also performed using lysates of purified macrophage-grown PRRSV (Fig. 4). The results showed that also the M/GP_5_ glycoprotein complex of macrophage-grown PRRSV binds to the pSn4D-Fc protein. Binding was also critically dependent on the sialic acid-binding capacity of pSn, as no interaction was observed with the non-sialic acid-binding mutant pSn4D_RE_-Fc. No binding was observed for the GP_3_ and GP_4_ proteins. These results suggest that macrophage-grown and MARC-145-grown PRRSV interact with pSn in the same way.

## Discussion

PRRSV has a very restricted tropism for subpopulations of differentiated cells of the monocyte/macrophage lineage. The virus infects macrophages in lungs and several lymphoid organs, while other cells such as circulating blood monocytes and peritoneal macrophages are refractory [53],[54]. This restricted cell tropism has been attributed to the restricted expression pattern of the PRRSV receptor pSn on these subsets of differentiated macrophages [36],[44],[55]. Since pSn is an essential PRRSV-binding and -internalization receptor on porcine macrophages, identification of the PRRSV glycoprotein ligands that mediate virus binding to this receptor is clearly of great importance, not only for basic understanding of PRRSV replication biology, but also for development of strategies to tackle PRRSV infection. For many viruses (e.g. classical swine fever virus, porcine circovirus 2), subunit vaccines comprising components involved in viral entry have proven very useful tools in the protection against viral infection [56]–[59]. A vaccine capable of inducing antibodies against the sialoadhesin-binding epitope of PRRSV should be capable of providing a good protection against PRRSV infection by neutralizing virus entry. Previously, the interaction of PRRSV with pSn was shown to be dependent on a functional sialic acid-binding domain on pSn [38] and on the presence of sialic acids on the virion [38],[39], but the sialic acid-carrying viral ligand was never identified. This study aimed to identify PRRSV glycoprotein ligands for the pSn receptor. For this purpose, a soluble pSn was constructed and characterized. This soluble receptor showed the same binding functionality as wild type pSn, as it showed sialic acid-binding activity and could bind PRRSV in a sialic acid-dependent manner. Via pull-down assays in which the soluble receptor was mixed with lysates of PRRSV, it was shown that the M/GP_5_ complex of the PRRS virus interacts with the pSn receptor. This interaction was clearly dependent on the sialic acid-binding capacity of pSn, as a non-sialic acid-binding mutant of the soluble pSn differing in only one amino acid was not able to bind M/GP_5_. Removal of sialic acids from the virus prior to the pull-down assay also blocked the interaction of the M/GP_5_ complex with pSn, evidencing the importance of sialic acids on the GP_5_ protein for interaction with the receptor. Clearly, the characteristics of the interaction of M/GP_5_ with pSn are identical to these of the interaction of PRRSV particles with pSn, suggesting that the M/GP_5_ complex indeed mediates PRRSV-particle binding to pSn.

In this study, the interaction of PRRSV with the pSn receptor was initially studied using MARC-145-grown virus, as it is easy to obtain relatively high virus titers in these cells and it avoids the necessity of animal sacrifice. Moreover, MARC-145-grown virus can infect porcine alveolar macrophages and has been shown to interact efficiently with the pSn receptor [38],[40], indicating that MARC-145 cells provide correct glycosylation for the virus to interact with pSn. However, the glycome of the primary porcine macrophage, and hence of the virus grown therein, is most likely very different from the MARC-145 cell glycome. In the light of lectin-glycoprotein interaction, it was therefore imperative to evaluate the interaction of the viral glycoprotein(complexe)s of macrophage-grown PRRSV with the pSn receptor. Via pull-down experiments, in which lysates of macrophage-grown PRRSV were applied to immobilized recombinant sialoadhesins, it was found that also macrophage-grown PRRSV interacts with pSn via the M/GP_5_ glycoprotein complex. The interaction was also critically dependent on the sialic acid-binding capacity of pSn, indicating that the interaction of pSn with sialic acids is central to the pSn-M/GP_5_ interaction. While it should be kept in mind that primary macrophages cultured *in vitro* may also show different glycosylation than they do *in vivo*, it is clear that the interaction of pSn with macrophage-grown virus is more relevant to the *in vivo* situation than the interaction with MARC-145-grown virus.

Previous studies on PRRSV have shown that virus glycosylation is crucial for its infectivity towards macrophages. Treatment of the virus with N-glycosidase F to remove N-glycans has a negative impact on viral infectivity and removal of sialic acids from the virion surface using sialidase even results in a 10- to 20-fold reduction of the infectivity [39]. Considering the findings of the present study, these results suggest a central role of sialic acid-carrying N-glycans on the GP_5_ glycoprotein in the interaction with sialoadhesin. The GP_5_ protein of most PRRSV isolates contains two conserved N-glycosylation sites. One or more additional putative N-glycosylation sites can be present, depending on the virus isolate [25],[60],[61]. One of the conserved glycosylation sites, N^46^ in European isolates, N^44^ in American isolates, is particularly interesting in the light of the present study as it appears to be critical for the formation of infectious PRRSV virions [62],[63]. Wissink and coworkers showed that N^46^ is important for virus infectivity towards macrophages: the specific infectivity of recombinant GP_5_-N^46^Q mutant virus, in which the N^46^ glycosylation site was deleted, was reduced 10- to 20-fold when compared to the specific infectivity of wild type virus [62]. This correlates with the reduction in infectivity seen when virus is treated with sialidase to remove sialic acids from the surface [39]. Together, these data suggest that the critical sialic acid, necessary for M/GP_5_ binding to the pSn receptor, is present on the N-glycan appended to the N^46^/N^44^ amino acid. Another interesting observation is that the N^46^/N^44^ residue is part of the primary neutralization epitope of PRRSV [64]–[66]. While some other segments of the GP_5_ protein appear to be hypervariable, this epitope is located in an area of the GP_5_ ectodomain that is highly conserved among PRRSV isolates [65]–[70]. This suggests that this domain has a critical function in virus replication. It has been suggested to be crucial for the disulfide linkage between GP_5_ and M, as the cysteine residue involved in this linkage lies within the conserved area and since the linkage between GP_5_ and M is critical for virion formation [23],[26]. It is however also tempting to speculate that this critical function lies in the interaction of the GP_5_ glycoprotein with the sialoadhesin receptor, this via essential sialic acids present on the glycan appended to the N^46^/N^44^ residue of GP_5_. This could also explain why antibodies directed against this epitope appear to have a neutralizing effect on infection of macrophages. Previously, a study by Delputte *et al.* showed that neutralizing antibodies can indeed block attachment and internalization of the virus into macrophages [71].

Sialic acids are acidic monosaccharides often present at the termini of glycan chains on animal glycoconjugates [72]. Siglecs like sialoadhesin display a marked preference for specific types of sialic acids. However, also the linkage of the sialic acid to the subterminal sugar residue seems to influence the affinity of a siglec for a specific ligand. In addition, formation of high affinity interactions can depend on additional structural features of the glycoconjugate that carries the sialic acid [73]. These factors contribute to the specificity of a siglec for specific sialylated ligands and may explain why the GP_3_ and GP_4_ envelope proteins, although they carry sialic acids, do not seem to interact with sialoadhesin, while the M/GP_5_ glycoprotein complex shows a strong binding to this receptor. Furthermore, it is well known that physiologically relevant, high affinity lectin-glycan interactions strongly depend on the lectin and glycan valency [74]. As the pSn receptor is abundantly expressed at the macrophage cell surface [36] and as a single PRRSV virion carries a whole array of M/GP_5_ complexes on its surface [75], it can be expected that also the interaction between macrophage and virus depends on avidity rather than on simple affinity.

In conclusion, we identified the M/GP_5_ complex of PRRSV as a ligand for the pSn receptor. Furthermore, we showed that the M/GP_5_-pSn interaction is critically dependent on the sialic acid-binding capacity of pSn as well as on sialic acids on the viral GP_5_ glycoprotein. The information and tools generated in this study can provide a theoretical and practical basis for the development and evaluation of a new generation of inactivated and subunit PRRSV vaccines.

## Supporting Information

Protocol S1 Immunofluorescence staining and confocal microscopy
(0.04 MB PDF)

Protocol S2 Fc- and pSn-specific ELISA assays
(0.06 MB PDF)
