# Supplementary material for: The M/GP5 Glycoprotein Complex of Porcine Reproductive and Respiratory Syndrome Virus Binds the Sialoadhesin Receptor in a Sialic Acid-Dependent Manner
Source: PLoS Pathog. 2010 Jan 15;6(1):e1000730. doi: 10.1371/journal.ppat.1000730 (PMC2799551; doi:10.1371/journal.ppat.1000730)
Supplement: Protocol S1 — Immunofluorescence staining and confocal microscopy (0.04 MB PDF) [file ppat.1000730.s001.pdf]

HEK-293T cells were transfected with the vectors encoding the pSn-Fc chimeras using Lipofectamine (Invitrogen; manufacturer's instructions were followed) and fixed with 3 % paraformaldehyde 24 h after transfection. Cells were permeabilized with 0.1 % Triton X-100, washed and incubated with pSn-specific mAb 41D3, followed by incubation with Texas Red-labeled goat anti-mouse antibodies (Molecular Probes). Subsequently, they were stained with FITC-labeled goat anti-human IgG Fc-specific antibodies (Sigma-Aldrich Corp.). In between different incubation steps, cells were washed 3 times with PBS. Finally, cells were washed, embedded in a glycerine-PBS solution (0.9/0.1 v/v) containing 2.5 % 1,4-diazabicyclo(2,2,2)octane, mounted and analyzed using a TCS SP2 laser scanning spectral confocal system (Leica Microsystems) using an Argon 488 nm and a Gre/Ne 543 nm laser for excitation.
