# Supplementary material for: The M/GP5 Glycoprotein Complex of Porcine Reproductive and Respiratory Syndrome Virus Binds the Sialoadhesin Receptor in a Sialic Acid-Dependent Manner
Source: PLoS Pathog. 2010 Jan 15;6(1):e1000730. doi: 10.1371/journal.ppat.1000730 (PMC2799551; doi:10.1371/journal.ppat.1000730)
Supplement: Protocol S2 — Fc- and pSn-specific ELISA assays (0.06 MB PDF) [file ppat.1000730.s002.pdf]

Each well of an Immulon 4HBX 96-well flat-bottomed microtiter plate (Dynax Technologies Inc.) was coated with 50 µl of 8 µg/ml goat anti-human IgG (Fc-specific; Sigma-Aldrich Corp.) in 0.05 M carbonate-bicarbonate buffer (pH 9.6; Sigma-Aldrich Corp.). Plates were washed with PBS containing 0.25 % bovine serum albumin (PBA), after which 2-fold dilution series of the purified siglec-Fc chimera were added to the wells and incubated for 2 h at ambient temperature. Unbound siglec-Fc protein was removed by washing with PBA.

Fc-specific ELISA

50 µl alkaline phosphatase-conjugated goat anti-human IgG (Fc-specific; Sigma-Aldrich Corp.) diluted 1:5000 in PBA was added to each well and incubated for 1 h at room temperature. The plate was washed 4 times with Tris-buffered saline containing 0.25 % bovine serum albumin (TBA) and 100 µl of 5 µM fluorescein diphosphate (FDP; Molecular Probes) in substrate buffer (100 mM Tris, 100 mM NaCl, 5mM $MgCl_2.6H_2O$; pH 9.5) was added to each well. Fluorescence at 535 nm was measured using a Cytofluor multi-well plate reader (PerSeptive Biosystems) with the excitation and emission wavelengths of 485 nm and 535 nm, respectively.

pSn-specific ELISA

100 µl mAb 41D3 (50 µg/ml in PBA) was added to each well and incubated for 1 h at 4 °C. After washing with PBA, 50 µl alkaline phosphatase-conjugated goat anti-mouse IgG (Fc-specific; Sigma-Aldrich Corp.) diluted 1:2500 in PBA was added to each well and incubated for 1 h at room temperature. The plate was then washed 4 times with TBA, 100 µl of 5 µM FDP in substrate buffer was added to each well and fluorescence was measured as described above.
